# Outcomes of Free Flap Surgery in Head and Neck Cancer Patients With Chronic Kidney Disease: A Single-Institution Retrospective Study of 61 Cases

**DOI:** 10.7759/cureus.98897

**Published:** 2025-12-10

**Authors:** Keitaro Nagano, Masami Osaki, Kiyomi Kuba, Akio Hatanaka, Mutsuko Hara, Kazue Manaka, Shingo Kinoshita, Kazuhiro Mitsumura, Ryohei Mukae, Eisuke Ohtake

**Affiliations:** 1 Department of Otolaryngology - Head and Neck Surgery, Ageo Central General Hospital, Ageo, JPN

**Keywords:** acute renal failure and hemodialysis in icu, anastomosed vessel pathology, chronic kidney disease (ckd), continuous hemodiafiltration, flap necrosis, head and neck cancer (hnc), head and neck surgery with free flap reconstruction, postoperative complications, ­reconstructive surgery, thrombosis

## Abstract

Introduction

The number of head and neck cancer patients with chronic kidney disease (CKD) is increasing. Although free flap reconstruction has become more feasible with advances in renal replacement therapy, perioperative risks in CKD remain uncertain. Prior reports are limited and inconclusive. We evaluated free flap outcomes in CKD patients undergoing head and neck reconstruction.

Materials and methods

We performed a single-institution retrospective analysis of 61 patients with CKD, operationalized by preoperative estimated glomerular filtration rate (eGFR) within the presurgical window (<60 mL/min/1.73 m²), who underwent free flap reconstruction (2011-2022). The primary outcome was flap complications (postoperative thrombosis requiring thrombectomy or flap necrosis). Failure cases underwent histopathologic review of anastomosed vessels. A prespecified descriptive comparison contrasted flap events by diabetes mellitus (DM) status. Statistical analysis used Fisher’s exact test; owing to only five events, no multivariable models were fitted. Postoperative continuous hemodiafiltration (CHDF) use was recorded.

Results

The most common eGFR category was G3a (65.1%), followed by G3b, G5, and G4. Flap complications occurred in 5/61 (8.2%) patients: necrosis (3/61; 4.9%) and thrombosis salvaged by thrombectomy (2/61; 3.3%). All events arose in G3a; no events were seen in G3b-G5 or among CHDF cases. Histopathology showed severe occlusive change in one necrosis case (~80% luminal stenosis), while other failures had at most mild intimal thickening; two failures occurred without diabetes mellitus (DM)/cardiovascular disease or evident vascular pathology. DM was present in 14/61 (23%) patients; event proportion was 14.3% with DM vs. 6.4% without DM (Fisher p = 0.32), a non-significant difference consistent in direction with prior reports.

Discussion

Events were infrequent and clustered in G3a; the absence of events in higher categories is not interpretable, given very small subgroup sizes and limited power. Vascular pathology among failures was heterogeneous, indicating that overt vasculopathy or glycemic status alone does not fully explain failure. Careful perioperative management may support flap viability, but causal inference is unwarranted.

Conclusion

Free flap reconstruction appears feasible in selected CKD patients with optimized perioperative care; however, findings are hypothesis-generating and require confirmation in larger controlled cohorts with standardized operative reporting and adjustment for confounding.

## Introduction

Chronic kidney disease (CKD) is a growing global health concern, with its prevalence rising among patients requiring complex head and neck cancer reconstruction [1]. Advances in renal replacement therapy have expanded surgical options, including microvascular free flap procedures, yet perioperative risk stratification in CKD remains challenging and clinically significant. Recent multicenter and matched cohort studies demonstrate that patients with advanced CKD (G4/5) are at increased risk for free flap failure and overall postoperative complications, with risk escalating alongside CKD severity [2]. In contrast, some evidence suggests that end-stage renal disease (ESRD) patients on dialysis do not experience higher rates of flap failure, though they may have longer intensive care stays and a greater burden of comorbidities [3,4].

Comorbidity burden, particularly diabetes mellitus, malnutrition, and cardiovascular disease, is consistently identified as a major predictor of both medical and surgical complications, including flap necrosis and wound infection [5-9]. Perioperative factors such as prolonged operative time, transfusion requirements, and intraoperative fluid management further influence complication rates [3,8,10,11]. Optimization of nutritional status, albumin levels, and structured perioperative care pathways, such as enhanced recovery after surgery (ERAS) protocols, have demonstrated reductions in hospital length of stay and wound complications, though overall complication rates remain substantial [9,12,13].

Technical factors, including the choice of venous anastomosis, also play a critical role in flap survival, sometimes outweighing patient characteristics [14]. In patients with multimorbidity, simplified reconstructive approaches may be warranted to balance functional outcomes with perioperative risk [15].

We aimed to (i) evaluate flap complications by preoperative eGFR category, (ii) record postoperative continuous hemodiafiltration (CHDF) use, and (iii) describe histopathologic features of anastomosed vessels in failure cases. Our prespecified primary hypothesis was that lower preoperative eGFR categories would be associated with a higher free flap failure rate. Secondary outcomes were CHDF use, other major postoperative complications, and descriptive histopathology in failures. To enhance reproducibility and cross-study comparability, we adopted standardized, a priori outcome definitions and a consistent reporting framework, in line with recommendations advocating standardized outcome reporting in surgical research [16]. Given the single-center design and low event counts, the analysis is primarily hypothesis-generating.

## Materials and methods

Study design and cohort

We retrospectively reviewed head and neck cancer patients with CKD who underwent microvascular free flap reconstruction at our institution between July 2011 and August 2022. For this retrospective cohort, CKD status was operationalized by preoperative renal function as detailed below. Inclusion criteria required: (i) histologically confirmed head and neck malignancy; (ii) free flap reconstruction; and (iii) at least one preoperative eGFR measured within 30 days of surgery. Exclusion was limited to insufficient records that precluded outcome ascertainment, including the absence of a preoperative eGFR within 30 days; cases with clear, isolated acute kidney injury (AKI) without persisting dysfunction, when clinically documented, were also excluded. All charts were reviewed for features suggestive of AKI (e.g., creatinine dynamics consistent with the KDIGO (Kidney Disease: Improving Global Outcomes) AKI criteria [17] or documented acute illness); no patient met these criteria or raised a strong suspicion of AKI, and therefore no case was excluded on this basis. The risk of AKI-related misclassification was considered minimal for this cohort. Demographics, tumor stage, comorbidities (including diabetes mellitus (DM) and ischemic heart disease), cardiac function, intraoperative parameters, and postoperative course were extracted from medical records. DM was recorded based on documented diagnosis and/or antidiabetic therapy; HbA1c values closest to surgery were abstracted when available.

Renal function categorization

Because longitudinal (≥3 months) data to confirm chronicity were not available in this retrospective cohort, we categorized preoperative renal function by estimated glomerular filtration rate (eGFR) within the preoperative window (from initial evaluation to surgery). For each patient, we extracted all outpatient serum creatinine measurements in this window and assigned categories corresponding to KDIGO thresholds (G3a: 45-59; G3b: 30-44; G4: 15-29; G5: <15 mL/min/1.73 m²) using the median eGFR value across available tests (or the most recent value if only one was available). Throughout, we therefore refer to “preoperative eGFR categories” rather than strict “CKD stages,” and interpret associations accordingly.

Outcomes and definitions

The primary outcome was a prespecified composite of free flap failure: (1) postoperative thrombosis requiring thrombectomy (salvaged) or (2) flap necrosis causing flap loss or reoperation. Secondary outcomes included (a) the individual components of the composite, (b) postoperative CHDF, and (c) other major postoperative complications (as abstracted from medical records). As a prespecified descriptive comparison, flap-related events (thrombosis or necrosis) were contrasted between patients with and without DM using Fisher’s exact test. Outcome definitions were specified a priori and reported using a standardized framework to facilitate comparability and reduce reporting heterogeneity [16].

Operative team, flap selection, and anticoagulation strategy

Procedures were performed by a pair comprising an attending microvascular surgeon and a trainee (resident/fellow) in most cases. While trainees occasionally performed discrete steps under supervision, the attending surgeon was responsible for the majority of each operation and the final decisions. Retrospective chart review did not allow a clear-cut attribution of specific steps (e.g., anastomosis) to attending versus trainee on a per-case basis; therefore, surgeon-level stratification was not feasible.

Flap type and pedicle selection followed standard reconstructive principles (defect-driven geometry and vessel quality/availability). Given the small sample size, we did not model outcomes by detailed flap geometry/pedicle variables; however, flap type was specified in all failure cases to aid interpretability. No routine intraoperative systemic anticoagulation or antiplatelet therapy was used. Standard topical hemostasis and meticulous microvascular technique were applied.

Anesthesia and perioperative management

Anesthesia and fluid management were goal-directed with the aims of optimizing cardiac output and oxygen delivery to end organs and the flap while avoiding volume overload. Hemodynamics and urine output were monitored continuously; transfusion was considered early when clinically indicated, consistent with the renal-protection strategy. Given the potential role of inflammation in AKI, for long-duration ablative and reconstructive head-and-neck free-flap surgeries, a single intravenous pre-incision dose of dexamethasone (6.6-9.9 mg; reflecting the 3.3 mg and 6.6 mg formulations available in Japan) was administered within 30 minutes before skin incision; no postoperative taper was used, although evidence specific to AKI prevention remains limited [18-21].

Histopathology in failure cases

In cases of flap failure, arterial and/or venous segments from the anastomosis/pedicle were examined on hematoxylin and eosin (H&E) by two board-certified pathologists blinded to the clinical course and outcomes. Findings were recorded qualitatively using a semi-structured template, including the presence and apparent severity of intimal thickening/hyperplasia, the approximate degree of luminal narrowing, the presence and character of thrombus (acute vs. organized), endothelial injury/denudation, inflammatory change, and calcification. No formal quantitative grading scale was applied. Discrepancies were resolved by joint review. This explicit procedural description follows recommendations to improve measurement reproducibility in surgical research [22] and aligns with standardized outcome reporting [16].

Statistical analysis

Categorical variables were summarized as counts and percentages. Fisher’s exact test (two-sided, α = 0.05) was used for comparisons. Analyses were stratified by preoperative eGFR category. A prespecified subgroup contrast for flap-related events by diabetes status was performed using Fisher’s exact test. Because only five flap-event cases occurred, multivariable modeling was not attempted. Where referenced, the institutional overall rate for flap necrosis during the same period is reported for context rather than for direct inference to non-CKD subgroups. All analyses were conducted with standard statistical software.

Ethics

This study adhered to ethical guidelines and was approved by the hospital’s ethics committee (registration number: 1329). Data collection and analysis followed all institutional and ethical standards, and patient confidentiality was maintained throughout the study.

## Results

Of the screened patients, none were excluded for missing data; all eligible cases were included in the analysis. A total of 61 of 228 patients (26.8%) met the CKD criteria and were analyzed. The mean age was 72 years (range, 50-87), with 51 males and 10 females. Most had advanced-stage disease (n = 58), and the mean left ventricular ejection fraction (LVEF) was 59% (range, 42-78%) (Table 1).

**Table 1:** Patient characteristics (n = 61). Values are presented as n (%), unless otherwise indicated. Age and left ventricular ejection fraction (LVEF) are shown as mean (range).

Characteristics	Value
Mean age	72 (50-87)
Gender	
Male	51 (83.6)
Female	10 (16.4)
Clinical stage (Stage III/IV)	58 (95.1)
Left ventricular ejection fraction (LVEF)	59.3 (42-78)
Diabetes mellitus	14 (23.0)
Cardiovascular disease	7 (11.4)

Regarding renal function, G3a was the most common stage, followed by G3b, G5, and G4 (41, 16, 4, and 2 cases, respectively). Three of the four patients in G5 were on maintenance hemodialysis prior to surgery, and one patient in G5 required initiation of hemodialysis postoperatively. CHDF was performed in one G4 and one G5 patient (Table 2).

**Table 2:** Distribution of patients by eGFR category and requirement for CHDF. Column 2: n (% of total, N = 61). Column 3 (“Postoperative CHDF, n”) counts patients who started CHDF after surgery (excludes those on preoperative maintenance hemodialysis). Perioperatively, two patients required CHDF (G4: n = 1; G5: n = 1). In G5, three were on maintenance hemodialysis preoperatively, and one initiated maintenance hemodialysis postoperatively. CHDF: continuous hemodiafiltration; eGFR: estimated glomerular filtration rate.

eGFR categories	n (%)	Postoperative CHDF, n
G3a (eGFR: 45-59)	39 (63.9)	0
G3b (eGFR: 30-44)	16 (26.2)	0
G4 (eGFR: 15-29)	2 (3.2)	1
G5 (eGFR: <15)	4 (6.5)	1

Both CHDF cases had undergone total pharyngolaryngoesophagectomy, bilateral neck dissection, and free jejunal reconstruction. The first CHDF case was a 78-year-old male (G5) with preoperative creatinine of 4.07 mg/dL and eGFR of 10.5 mL/min/1.73 m², and an ejection fraction (EF) of 58%. Intraoperatively, he developed oliguria (total urine output = 90 mL) with a positive fluid balance of +3510 mL, leading to initiation of CHDF starting immediately after surgery. The second CHDF case was a 73-year-old male (G4) with preoperative creatinine of 2.20 mg/dL, eGFR of 23.9 mL/min/1.73 m², and EF of 64%. An abdominal aortic aneurysm was diagnosed preoperatively, and after consultation with the cardiovascular surgery team, the patient underwent cancer surgery with their standby support. On postoperative day two, the aneurysm ruptured below the renal artery, requiring emergency surgery and CHDF for two days due to oliguria from hypovolemic shock. Both patients were eventually discharged without further complications.

Flap complications occurred in 5/61 (8.2%) cases, with flap necrosis in 3/61 (4.9%) and thrombosis salvaged by thrombectomy in 2/61 (3.3%), and all events occurred in CKD G3a. No flap complications were observed in G3b, G4, or G5, including the two CHDF cases (Table 3).

**Table 3:** Incidence of flap vascular complications by eGFR category. Values are presented as n (% of total, N = 61), unless otherwise indicated. “Flap vascular complications” comprise postoperative thrombosis requiring thrombectomy and flap necrosis. eGFR: estimated glomerular filtration rate.

eGFR category	n (%)	Flap vascular complications, n	Thrombectomy (salvaged), n	Flap necrosis, n
G3a	41 (65.1)	5	2	3
G3b	16 (25.4)	0	0	0
G4	2 (3.2)	0	0	0
G5	4 (6.3)	0	0	0

DM was present in 14/61 (23%) patients. Flap-related events occurred in 2/14 (14.3%) with DM vs. 3/47 (6.4%) without DM (odds ratio: 2.44; 95% CI: 0.37-16.34; Fisher’s exact p = 0.32). Owing to the limited number of flap events (n = 5), no multivariable models were fitted; accordingly, these subgroup comparisons are descriptive.

Among the three cases of flap necrosis, two involved anterolateral thigh (ALT) flaps and one involved a rectus abdominis flap. Of the two ALT cases, one patient had no significant medical history, while the other had DM (HbA1c = 6.7%) and ischemic heart disease. The rectus abdominis case also involved diabetes (HbA1c = 7.8%) and ischemic heart disease. Regarding the two thrombectomy cases, one was an ALT flap in a patient with no major comorbidities, and the other was a radial forearm flap in a patient with a history of transient ischemic attack (TIA) as the only comorbidity. No major cardiovascular or other postoperative complications were observed other than the flap-related issues.

Among five failure cases, severely compromised vascular status was identified in only one necrosis case: pronounced intimal hyperplasia with ~80% luminal stenosis (HbA1c = 6.7%). In the remaining cases, including one necrosis (HbA1c of 7.8% with ischemic heart disease) and one thrombectomy (history of TIA), only mild fibrous intimal thickening was observed. Two failures occurred in patients without DM, cardiovascular disease, or evident vascular pathology.

## Discussion

Of the 228 reconstructive surgeries performed during the study period, 61 (26.8%) involved patients with CKD, mirroring the rising prevalence of CKD among surgical candidates. Flap events were uncommon and clustered in G3a; no events occurred in G3b-G5 or among patients who required postoperative CHDF. The flap necrosis rate in CKD was 4.92%, and when contextualized against our institutional rate of 2.91%, the difference was not statistically significant (Fisher’s exact test, p = 0.435). Histopathology identified severe vascular disease in only one necrosis case, whereas the remaining failures showed at most mild changes; notably, two failures occurred in patients without diabetes, cardiovascular disease, or evident vascular pathology, suggesting that overt vascular or glycemic pathology alone does not fully account for failure and that non-patient factors may contribute. Although our DM-stratified analysis was underpowered and not statistically significant, the observed direction (higher event proportion in DM) is consistent with prior reports identifying DM as a risk factor for flap complications. Larger cohorts are needed to quantify the independent effect after adjustment for confounders. This single-institution series with low event counts was underpowered for definitive inference; accordingly, the results should be viewed as hypothesis-generating despite the prespecified primary endpoint and standardized reporting.

From a perioperative management standpoint, preventing AKI requires particular attention to two major factors: renal hypoperfusion and systemic inflammation [23-25]. For patients with renal anemia (hemoglobin <8.0 g/dL), preoperative transfusion may help maintain adequate renal perfusion; intraoperative blood loss should be closely monitored, with early transfusion considered when indicated. Volume overload should be avoided, as venous congestion can worsen renal hypoperfusion and thereby increase AKI risk. In patients with CKD, even modest volume overload can precipitate clinically significant lung congestion [26]. Regarding inflammation, selective preoperative steroid use, aimed at blunting cytokine-mediated endothelial injury, has been associated with fewer complications in esophageal surgery [23], though evidence for AKI prevention per se remains limited. In our practice, steroids are administered prior to highly invasive procedures to reduce overall complications and length of stay [24]. Alongside vigilant fluid management, these measures, together with an early initiation of CHDF policy for refractory oliguria, may have contributed to the low incidence of CKD-specific complications observed here; however, causality cannot be inferred.

Whether CKD itself increases the risk of free flap failure remains controversial. Some studies imply increased risk in advanced CKD, whereas others do not. For example, Inoue et al. reported only one case of flap necrosis among 10 hemodialysis patients [26]. In a larger cohort of 251 reconstructions, 19.5% developed necrosis, yet renal impairment was not an independent risk factor [27]. Likewise, Lin et al. found no association between impaired renal function and necrosis across 1,284 ALT reconstructions [28]. Consistent with these reports, our data do not support CKD stage ≥G3b or postoperative CHDF as signals of increased flap risk within our setting.

Limitations

This single-center, retrospective study had a modest sample size and low event counts, limiting statistical power. Potential misclassification of renal status at surgical planning and inter-case variability in perioperative protocols (fluid targets, transfusion thresholds, antithrombotic management) may affect interpretation. Over the 11-year period, perioperative protocols and operative practice likely evolved, which may limit strict comparability across eras. Long-term renal outcomes and late flap events were not systematically captured. Reproducibility is further constrained because surgeon-level attribution (attending vs. trainee) and detailed flap geometry/pedicle metrics could not be reliably abstracted from charts; to mitigate this, we report these elements narratively and specify flap type in failure cases. Stage-specific inference in G4-G5 is particularly unreliable due to very small numbers; findings in these strata should be considered hypothesis-generating. Because the small number of events precluded meaningful multivariable adjustment, residual confounding (e.g., diabetes burden, flap/pedicle nuances, and surgeon variability) remains a plausible explanation for some findings. Taken together, these issues underscore the value of standardized outcome definitions and procedural reporting to improve comparability and reduce bias, as advocated in methodological guidance [16,22,29,30]. Given these constraints, findings should be interpreted as hypothesis-generating, and larger multi-center prospective studies with standardized, trial-like operative reporting are needed to validate and extend these observations.

## Conclusions

In this single-center series of 61 CKD patients undergoing head and neck free flap reconstruction, flap events were infrequent and clustered in G3a; the absence of events in G3b-G5 and in CHDF cases should be interpreted cautiously, given very small subgroup sizes. Histopathology in failures was heterogeneous, with severe occlusive change in only one case, suggesting that overt vascular or glycemic pathology alone does not fully explain failure. The institutional comparison was descriptive and underpowered, so statements about risk differences are not warranted. Overall, these findings are hypothesis-generating and support the need for larger, controlled cohorts with standardized perioperative reporting and adjustment for confounding.
